# Effectiveness of the Original COVID-19 Vaccine against COVID-19 Exacerbations during the Omicron Wave: A Population-based Study in Okayama, Japan

**DOI:** 10.31662/jmaj.2023-0019

**Published:** 2023-09-27

**Authors:** Naomi Matsumoto, Toshiharu Mitsuhashi, Rumi Matsuo, Tomoka Kadowaki, Soshi Takao, Takashi Yorifuji

**Affiliations:** 1 Department of Epidemiology, Faculty of Medicine, Dentistry and Pharmaceutical Sciences, Okayama University, Okayama, Japan; 2 Center for Innovative Clinical Medicine, Okayama University Hospital, Okayama, Japan; 3 Department of Epidemiology, Graduate School of Medicine, Dentistry and Pharmaceutical Sciences, Okayama University, Okayama, Japan

**Keywords:** COVID-19, Vaccine, Omicron, Prevention, Pneumonia

## Abstract

**Introduction:**

In Japan, approximately 97 million individuals have received their primary two doses of coronavirus disease 2019 (COVID-19) vaccine at the end of 2022. In this study, we aim to examine the effectiveness of the primary vaccines and compare its efficacy to booster vaccine shots in terms of preventing COVID-19 exacerbations during the Omicron-predominant period in Japan.

**Methods:**

For this analysis, we have collected all the confirmed COVID-19-positive cases from different medical institutions in Okayama City and have also utilized the information from the public Vaccination Record System. Taking the number of vaccinations into consideration, we then conducted a population-based study to assess the effectiveness of the two primary vaccine doses in preventing COVID-19 exacerbations during the Omicron waves. Our primary and secondary outcomes were COVID-19 exacerbations with respiratory failure (i.e., oxygen saturation on room air ≤ 93%, requiring supplemental oxygen), intensive care unit admission and/or mechanical ventilator requirement, or death, in accordance with the Japanese COVID-19 guidelines, and pneumonia during the course of COVID-19 infection, respectively.

**Results:**

In total, 95,329 COVID-19-positive individuals, aged 5 years and above, were included in this analysis (study period from January 1 to September 10, 2022). As per our findings, the effectiveness of the primary two doses against COVID-19 exacerbations compared with those who had never been vaccinated was 55.5% (95% confidential interval [CI]: 32.6-71.7), whereas it was higher after the third dose (76.9%; 95% CI: 66.7-84.0) and the fourth dose (75.7%; 95% CI: 58.8-85.7). Effectiveness was sustained for ≥ 5 months after the third vaccination, and preventive effectiveness was observed in individuals aged ≥ 65 years.

**Conclusions:**

As per the results of this study, we can conclude that the efficacy of the primary two doses of SARS-CoV-2 vaccine can be further strengthened in terms of preventing COVID-19 exacerbations by administering third and fourth booster vaccine shots. The additional bivalent vaccine is anticipated to further increase its efficacy against the Omicron strain, suggesting that individuals who have not received their booster shots yet should consider getting them to prevent COVID-19 exacerbations.

## Introduction

As of December 22, 2022, a cumulative total of 27,765,782 positive cases and 54,365 deaths caused by severe acute respiratory syndrome coronavirus 2 (SARS-CoV-2) viral infection was reported in Japan ^(1)^. In December of 2020, coronavirus disease 2019 (COVID-19) vaccines were introduced in Japan and demonstrated considerable efficacy of greater than 90% in terms of preventing infection, symptoms, and severe illness against the original coronavirus strains ^(2), (3), (4)^. However, the effectiveness of the primary two-dose vaccine series has declined over time, and the emergence of variants has led to the recommendation of booster shots. While the primary two-dose vaccine remained effective in preventing symptoms and severe diseases caused by the Delta strain prevalent in late 2021 ^(5)^, its effectiveness against the Omicron strain B.1.1.529 that became predominant in 2022 has reportedly attenuated ^(6), (7)^. Thus, an additional bivalent vaccine against the Omicron strain was launched in Japan on September 20, 2022. While reports demonstrating the effectiveness of the primary two-dose vaccine in preventing COVID-19 exacerbations during the Omicron wave can be sporadically found worldwide ^(6), (7), (8), (9)^, there are only a few reports specifically from Japan.

As of December 20, 2022, COVID-19 vaccine coverage in the Japanese population was reported to be at 80.4% for the primary two-dose regimen and 67.5% and 41.7% for the third and fourth doses, respectively ^(10)^. Approximately 20 million people have yet to receive their additional vaccinations after the primary two-dose series. It is thus needed to examine the effectiveness of the original two vaccine doses as well as additional doses in preventing severe disease, considering the number of shots given and the number of days since the last shot. This will provide important information for appropriate decision-making on whether individuals should receive further shots.

Therefore, we examined the effectiveness of the primary two-dose vaccine series and additional vaccines in preventing severe COVID-19 during the Omicron wave by analyzing the data on all positive patients residing in Okayama City and information from the official Vaccination Record System (VRS).

## Materials and Methods

### Study design and participants

This was a population-based study of all SARS-CoV-2-positive residents, aged 5 years or above, in Okayama City during the Omicron wave. In Japan, the surveillance of SARS-CoV-2 infections was based on mandatory reporting of laboratory-confirmed (polymerase chain reaction or antigen test) cases to the Health Center Real-time Information-sharing System on COVID-19. The extent of testing during the study period may vary depending on the prevailing epidemiological situation, but in general, symptomatic individuals, all close contacts of confirmed COVID-19 cases, and all personnel in facilities where clusters occurred were subjected to testing, irrespective of the presence of symptoms. Omicron strains were detected in 96.3% (BA. 1, 40.7%; BA. 2, 30.5%; BA. 4, 0.4%; BA. 5, 24.6%; Delta, 3.0%; undetectable samples, 0.8%) of the 2,362 genome-sequenced samples in Okayama City from December 28, 2021, to October 6, 2022 ^(11)^.

**Figure 1.**
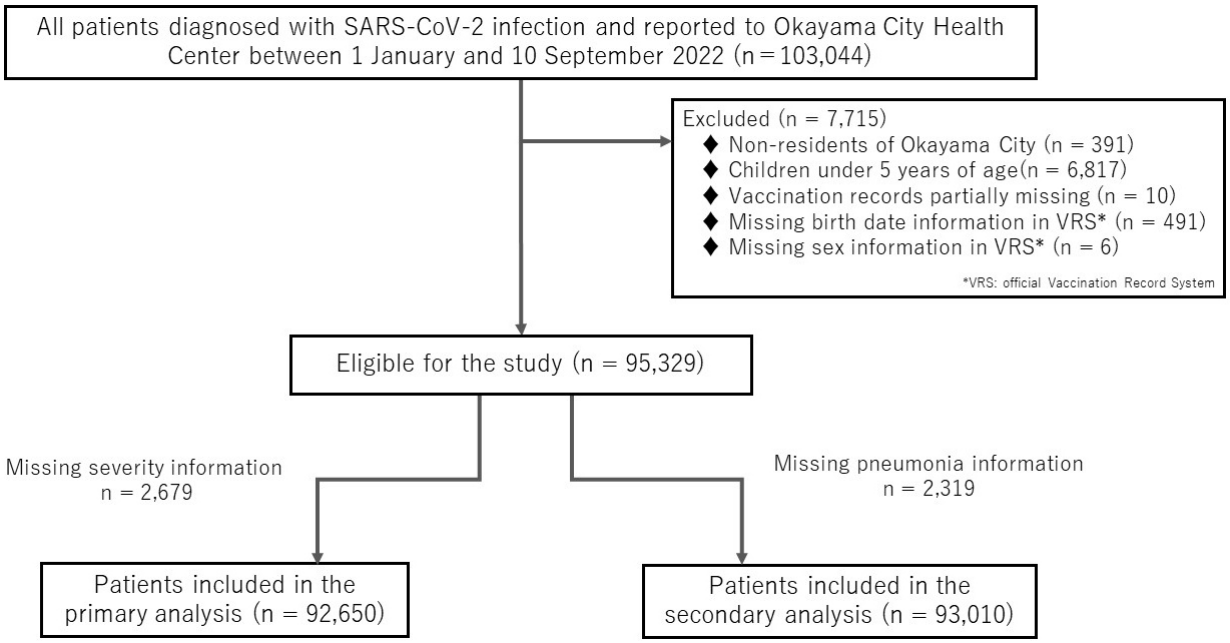
Flowchart of the study.

After September 26, 2022, incident reports were simplified to target high-risk cases only, which caused a surge in the number of unreported or untraceable positive cases. Therefore, we assessed the effectiveness of the primary two-dose vaccine against severe COVID-19 infection by examining the 95,329 of the 103,044 positive patients reported in Okayama City from January 1 to September 10, 2022 during the Omicron-predominant period. The decrease in the total number is attributed to the exclusion of individuals with missing crucial information, as indicated in Figure 1. In this study, we used administrative information on SARS-CoV-2-positive individuals residing in Okayama City, along with official vaccination records that include data on sex and date of birth, in cooperation with the Okayama City Public Health Center. As part of the data collection process, each dataset was linked to the public authorities, to ensure the records belong to the same individual. Subsequently, the provided data were anonymized, with personal information such as names and addresses removed to protect individuals’ privacy and confidentiality.

### Vaccination status

In Okayama City, the initial vaccinations against COVID-19 started in February 2021. As of September 13, 2022, 78.3% (551,874/705,000) of the total population had received their primary two-dose vaccination, whereas 61.4% (433,030/705,000) and 23.2% (163,391/705,000) had received their third and fourth doses, respectively. Detailed vaccination status by age group is provided in S-Table 1. All patients were vaccinated with mRNA-1273 or BNT162b2 vaccines because the bivalent vaccine against the Omicron strain was not administered until September 28, 2022 ^(12)^.

Data on vaccination date, the number of shots, vaccine type, and date of birth of the participants examined were obtained using the information from the VRS. The number of days that had passed after the last vaccination was calculated by subtracting the date of the last vaccination from the date of the COVID-19 diagnosis (the date of the positive test result). Vaccination was defined as completed when at least 7 days had passed since the last vaccination date.

### COVID-19 prognosis

The primary outcome was COVID-19 exacerbations with respiratory failure (i.e., oxygen saturation on room air ≤ 93%, requiring supplemental oxygen), intensive care unit admission and/or mechanical ventilator requirement, or death, in accordance with the COVID-19 guidelines in Japan ^(13)^. The secondary outcome was COVID-19 pneumonia detected via diagnostic imaging. Information regarding the outcome was obtained from the Okayama City Public Health Center’s COVID-19 patient records. The standard observation period for COVID-19 prognosis varied over time, starting with 10 days after the date of symptom onset or testing positive and then to 7 days. However, in cases where symptomatic individuals remained ill at the end of the standard observation period, the observation period was appropriately extended for continuous monitoring of their condition, including hospitalization and subsequent medical interventions due to worsening symptoms. Additionally, for individuals with multiple positive test results, the observation period was based on the date of symptom onset or the date of the first positive report.

### Covariates

Age was calculated by subtracting the date of birth from the date of diagnosis. Information on the date of birth and sex was included in the VRS. Information on comorbidities, obesity (i.e., body mass index ≥ 25), pregnancy, and smoking status was obtained from the Okayama City Public Health Center during the positive reporting process. Comorbidities included diabetes, respiratory disease, renal disease, liver disease, cardiac disease, hypertension, neuromuscular disease, hematologic disease, immunodeficiency, and malignancy. There were no missing values for comorbidities, obesity, and other relevant factors as they were considered “present” only if applicable.

### Statistical analysis

After describing the characteristics according to the number of vaccinations, Poisson regression with a robust error variance was conducted to determine any association between the number of vaccinations and primary and secondary outcomes. Risk ratios (RRs) and their 95% confidence intervals (CIs) were estimated by adjusting for age (age categories: 0-4, 5-11, 12-19, 20-29, 30-39, 40-49, 50-59, 60-64, and over 65 years), sex (dichotomous), presence of comorbidities (dichotomous), obesity (dichotomous), pregnancy (dichotomous), and smoking status (dichotomous), based on previous studies ^(14)^. Vaccine effectiveness was defined as (1−RR) × 100% ^(15)^.

A subgroup analysis was then performed by restricting the analysis to individuals aged 65 years and older, which are identified to be at high risk for COVID-19 exacerbations. Furthermore, using the information on the number of postvaccination days, we further divided individuals who received the third dose into two groups―“less than 150 days after vaccination” and “more than 150 days after vaccination”―and examined whether vaccine effectiveness in preventing COVID-19 exacerbations differed by the number of days after vaccination.

This study was approved by the Okayama University Graduate School of Medicine, Dentistry and Pharmaceutical Sciences Ethical Committee (no. K2110-020). Moreover, the requirement for informed consent was waived due to the retrospective nature of this study, with participant anonymity assured.

## Results

Among the 95,329 COVID-19-positive patients (48.9% male; 51.1% female) examined in this study, 35,008 were unvaccinated; 730 have completed their first dose; 31,009 have received their second dose; 26,382 have obtained their third dose; and 2,200 have received their fourth dose. Those who had never been vaccinated were mostly teenagers and younger participants; meanwhile, 65.3% of those who have already received their fourth dose were 65 years or older. Those with underlying diseases accounted for 10.4% of the participants who have received their primary doses but did not receive additional doses. The mean number of days after vaccination for those who received the second dose was 220.57 days (7-511 days), suggesting that many participants are yet to receive vaccination despite being eligible for the third dose (Table 1).

**Table 1.** Characteristics of COVID-19-Positive Patients Included in the Analysis by Vaccination Status.

Characteristics		Overall	Number of Vaccinations Completed				
			None	1	2	3	4
		n = 95,329	n = 35,008	n = 730	n = 31,009	n = 26,382	n = 2,200
		no. (%)					
Sex	Male	46,596 (48.9)	18,807 (53.7)	364 (49.9)	15,348 (49.5)	11,176 (42.4)	901 (41.0)
Age category (years)	5-11	13,723 (13.4)	12,606 (30.1)	185 (25.3)	932 (3.0)	0 (0.0)	N.A.
	12-19	11,867 (11.6)	5,227 (12.5)	127 (17.4)	5,231 (16.9)	1,276 (4.8)	6 (0.3)
	20-29	17,096 (16.7)	6,724 (16.1)	158 (21.6)	6,423 (20.7)	3,732 (14.2)	59 (2.7)
	30-39	16,182 (15.8)	4,180 (10.0)	102 (14.0)	6,926 (22.3)	4,835 (18.3)	139 (6.3)
	40-49	15,491 (15.2)	3,054 (7.3)	66 (9.0)	6,175 (19.9)	5,974 (22.6)	222 (10.1)
	50-59	9,027 (8.8)	1,686 (4.0)	43 (5.9)	2,896 (9.3)	4,226 (16.0)	176 (8.0)
	60-64	2,895 (2.8)	389 (0.9)	6 (0.8)	719 (2.3)	1,620 (6.1)	161 (7.3)
	65+	9,048 (8.9)	1,142 (2.7)	43 (5.9)	1,707 (5.5)	4,719 (17.9)	1,437 (65.3)
Comorbidities	Yes	8,611 (9.0)	2,224 (6.4)	67 (9.2)	3,210 (10.4)	2,945 (11.2)	165 (7.5)
Obesity	Yes	7,796 (8.2)	2,146 (6.1)	59 (8.1)	3,702 (11.9)	1,847 (7.0)	42 (1.9)
Pregnancy	Yes	533 (0.6)	146 (0.4)	5 (0.7)	265 (0.9)	117 (0.4)	0 (0.0)
Smoking status	Yes	6,493 (6.8)	2,396 (6.8)	52 (7.1)	2,968 (9.6)	1,060 (4.0)	17 (0.8)
Vaccine type	BNT162b2 only	43,274 (45.4)	N.A.	639 (87.5)	25,493 (82.2)	15,973 (60.6)	1,169 (53.1)
	mRNA-1273 only	7,966 (8.4)	N.A.	78 (10.7)	5,460 (17.6)	2,419 (9.2)	9 (0.4)
	Others *	9,081 (9.5)	N.A.	13 (1.8)	56 (0.2)	7,990 (30.3)	1,022 (46.5)
Days elapsed since the vaccination	Mean (SD)	172.42 (7-538)	N.A.	146.71 (7-538)	220.57 (7-511)	128.88 (7-370)	24.27 (7-102)

*Others (vaccine type) indicate that the patient took a combination of different vaccines.

### Vaccine effectiveness against COVID-19 exacerbations

Vaccine effectiveness against COVID-19 exacerbations after SARS-CoV-2 infection, as compared with those who were never vaccinated, was 55.5% (95% CI: 32.6-71.7) for those who have completed their two primary doses of vaccination, whereas its effectiveness was determined to be 76.9% (95% CI: 66.7-84.0) and 75.7% (95% CI: 58.8-85.7) for those who have received their third and fourth doses, respectively. Similar vaccine effectiveness was observed in the analysis results restricted to high-risk patients aged 65 years and older (Table 2).

**Table 2.** Vaccine Effectiveness by Dose against Severe COVID-19.

		Severe COVID-19	
	Number of vaccinations	Patients	Vaccine effectiveness*
		n/total (%)	percent (95% CI)
All	0	54/34,097 (0.2%)	Reference
	1	2/705 (0.3%)	−3.5% [−301.7-73.4]
	2	37/30,134 (0.1%)	55.5% [32.6-71.7]
	3	57/25,540 (0.2%)	76.9% [66.7-84.0]
	4	19/2,174 (0.9%)	75.7% [58.8-85.7]
			
Over 65	0	50/953 (5.3%)	Reference
	1	2/37 (5.4%)	−4.6% [−308.0-73.2]
	2	35/1,418 (2.5%)	52.5% [27.2-69.0]
	3	54/4,391 (1.2%)	77.0% [66.4-84.2]
	4	19/1,414 (1.3%)	76.5% [60.1-86.2]

*Adjusted for age, sex, comorbidities, obesity, pregnancy, and smoking status. CI: confidence interval

### Vaccine effectiveness against COVID-19 pneumonia

As a secondary outcome, vaccine effectiveness by dose against developing COVID-19 pneumonia, as compared with those who were never vaccinated, was 34.7% (95% CI: 19.5-47.1) among those who received their primary two doses, whereas its effectiveness was noted to be at 77.6% (95% CI: 71.8-82.2) among those given three doses and 88.0% (95% CI: 79.3-93.1) among those given four doses, suggesting increasing effectiveness as the number of doses increased. Similar results were observed in the analysis restricted to high-risk patients aged 65 years and older (Table 3).

**Table 3.** Vaccine Effectiveness by Dose against COVID-19 Pneumonia.

		COVID-19 Pneumonia	
Age (years)	Number of vaccinations	Patients n/total (%)	Vaccine effectiveness* percent (95% CI)
All	0	150/34,211 (0.4%)	Reference
	1	5/708 (0.7%)	−3.1% [−141.0-55.9]
	2	179/30,308 (0.6%)	34.7% [19.5-47.1]
	3	126/25,610 (0.5%)	77.6% [71.8-82.2]
	4	14/2,173 (0.6%)	88.0% [79.3-93.1]
			
Over 65	0	93/1,012 (9.2%)	Reference
	1	3/38 (7.9%)	13.6% [−160.8-71.4]
	2	138/1,550 (8.9%)	12.5% [−12.6-32.0]
	3	110/4,453 (2.5%)	73.2% [65.0-79.4]
	4	14/1,413 (1.0%)	87.7% [78.6-93.0]

*Adjusted for age, sex, comorbidities, obesity, pregnancy, and smoking status. CI: confidence interval

### Supplementary analysis

During the target period, additional vaccine doses with an interval of at least 5 months after the previous vaccination were recommended ^(16)^. Therefore, supplementary analysis was conducted by dividing the number of days that elapsed after the primary two doses and the third vaccination into “less than 150 days” and “more than 150 days.”

Vaccine effectiveness against COVID-19 exacerbations was 29.1% (95%CI: −29.9-78.2) among individuals who elapsed “less than 150 days” after the primary two doses, while it was 56.8% (95%CI: 34.1-71.7) among those who exceeded “more than 150 days.” Furthermore, for the third vaccination, vaccine effectiveness against COVID-19 exacerbations was 75.9% (95% CI: 59.9-85.1) in individuals who elapsed “less than 150 days” and 77.4% (95% CI: 65.8-85.1) in those who exceeded “more than 150 days.”

With regard to COVID-19 pneumonia, vaccine effectiveness was 30.9% (95% CI: −19.3-60.0) among individuals who elapsed “less than 150 days” after the primary two doses and 34.5% (95% CI: 18.39-47.1) among those who exceeded “more than 150 days.” Moreover, after the third vaccination, vaccine effectiveness against COVID-19 pneumonia was 68.9% (95% CI: 59.3-76.3) in individuals who elapsed “less than 150 days” and 84.5% (95% CI: 78.7-88.6) in those who exceeded “more than 150 days.”

## Discussion

In this study, we examined the effectiveness of the original two vaccine doses against COVID-19 exacerbations during the pandemic’s Omicron wave. In total, 95,329 COVID-19-positive patients residing in Okayama City, aged 5 years and above, were investigated in this study; moreover, information from the VRS were also utilized. Vaccine effectiveness against COVID-19 exacerbations and pneumonia in individuals who have received their two primary vaccination doses, as compared with those who had never been vaccinated, was 55.5% and 34.7%, respectively; meanwhile, vaccine effectiveness was higher among those who have received their third (76.9% and 77.6%) or fourth (75.7% and 88.0%) vaccination. Vaccine effectiveness against pneumonia during the course of COVID-19 infection was noted to be higher among those who received their fourth vaccination as compared to those who had their third dose. No reduction in vaccine effectiveness against COVID-19 exacerbations and pneumonia was observed among those who received their third vaccination, even after 150 days from vaccination.

Worldwide, the effectiveness of primary two-dose vaccines has been reported to have declined against the Omicron strain B.1.1.529. For instance, the preventive effect of mRNA-1273 against Omicron strain infection was reported to be 44.0% within 3 months from the second vaccination, which is deemed lower than its effectiveness against the conventional strain. The preventive effect of mRNA-1273 on Omicron strain infection declined over time but temporarily recovered to 71.6% 2 months after the third vaccination. The hospitalization prevention effect, which was expected to be moderately sustained over time, was effectively maintained 105 days after the third vaccination; it remained at 85.3%-86.8% for those aged 65 years and older, while it was noted to be 67.4%-75.9% for those aged 18 to 64 years, which attenuated from that of the conventional strain ^(6), (7)^. Although an additional bivalent vaccine against the Omicron strain has been encouraged in late 2022 to strengthen the effectiveness of the primary two-dose vaccine ^(17)^. There are few reports available in Japan regarding its real-world effectiveness. This is partly due to the elimination of the obligation to report all cases.

Given the accuracy of records, this study examined two prevention outcomes―the prevention of COVID-19 exacerbations (moderate disease 2 or higher based on Japanese guidelines) and the prevention of pneumonia―rather than effectiveness in preventing hospitalization. As in previous studies, the effectiveness of the primary two-dose COVID-19 vaccine against COVID-19 exacerbations and pneumonia was maintained at greater than 75% among those who received at least their third dose, and no decrease in terms of effectiveness was observed 150 days after the third dose. The pneumonia-preventive effect was even greater in those who received their fourth dose.

Despite the concerns and reports worldwide about the reduced prophylactic effectiveness of the primary two-dose vaccine against the Omicron variants ^(18), (19), (20)^. There remain few reports evaluating the effectiveness of this vaccine series alone against COVID-19 exacerbations among the Japanese population during the Omicron wave. In a study conducted by Kawasuji et al., a significant increase in antibody titers after bivalent booster vaccination was noted among healthcare workers ^(21)^. However, the study by Kawasuji et al. had a limited sample size and did not examine vaccine effectiveness in preventing severe disease in the general population in Japanese real-world settings. By evaluating data on all positive cases during the target period in a specific region of Japan, our study provided evidence to determine whether those who have not received their third and fourth doses after the primary vaccination (approximately 20 million people in Japan at the end of 2022) should be given additional vaccine doses. Furthermore, vaccination records were linked to the official VRS, ensuring a high degree of information accuracy.

This study has its limitations. For one, only COVID-19-positive participants were included in the analysis; thus, the effectiveness of infection prevention could not be evaluated. Additionally, we did not obtain information on prior infection history, which could have been relevant to assess the impact of prior immunity on vaccine effectiveness. In this study, we used the most severe disease level (moderate disease 2 of higher) and pneumonia progression during the course of the disease as health outcomes rather than hospitalizations, a common measure used worldwide. Hospitalization can be influenced by social factors (and the like); meanwhile, the accuracy of our outcome information sufficiently warrants the rationale to set the outcomes of this study. However, it is essential to acknowledge the potential presence of residual confounding factors, such as personal infection prevention behavior and attitudes toward seeking medical care, which might have led to an overestimation of the vaccine’s effectiveness. This study could be susceptible to “healthy vaccinee bias,” where individuals who chose to get vaccinated might also engage in other preventive behaviors, which results in the overestimation of the vaccine’s effectiveness.

In summary, the primary two-dose SARS-CoV-2 vaccine demonstrated greater effectiveness against COVID-19 exacerbations in individuals who received their third or fourth vaccination as compared to those who only received their second dose. Efficacy was sustained for up to 5 months or more after the third vaccination, and preventive effectiveness was also observed in individuals aged 65 or older. The additional bivalent vaccine against the Omicron strain is expected to further enhance vaccine efficacy; thus, those who have not yet received their booster vaccines should consider doing so to prevent COVID-19 exacerbations.

## Article Information

### Conflicts of Interest

None

### Sources of Funding

This work was supported by Okayama City grant number 7402100053 for COVID-19 research projects.

### Acknowledgement

We thank Hiroaki Matsuoka, Tomoe Kodama, Saori Irie, and Yoko Oka for their valuable support in data collection. We also thank Anahid Pinchis from Edanz (https://jp.edanz.com/ac) for editing a draft of this manuscript.

### Author Contributions

NM analyzed the data and wrote the first draft. TM, RM, TK, ST, and TY contributed to the interpretation of the data and the revision of this manuscript. All authors have read and approved the final manuscript.

### Approval by Institutional Review Board (IRB)

This study was approved by the Ethical Committee of the Graduate School of Medicine, Dentistry and Pharmaceutical Sciences of Okayama University (no. K2110-020).

### Data Availability

Data cannot be shared for privacy or ethical reasons.

## Supplement

S-Table
