# Supplementary material for: Effectiveness of the Original COVID-19 Vaccine against COVID-19 Exacerbations during the Omicron Wave: A Population-based Study in Okayama, Japan
Source: JMA J. 2023 Sep 27;6(4):463–9. doi: 10.31662/jmaj.2023-0019 (PMC10628327; doi:10.31662/jmaj.2023-0019)
Supplement: S-Table [file 2433-3298-6-4-463-s001.pdf]

S-Table 1. Number of persons vaccinated with each vaccine dose by age group

	Total Population	Number of Vaccinations Completed			
		1	2	3	4
Age (years)	no.	no. (%)			
0–4	28,000	N.A.	N.A.	N.A.	N.A.
5–11	45,000	9,108 (20.2)	8,384 (18.6)	31 (0.1)	N.A.
12–19	53,000	40,343 (76.1)	39,934 (75.3)	21,528 (40.6)	150 (0.3)
20–29	77,000	62,209 (80.8)	61,527 (80.8)	37,895 (49.2)	3,252 (4.2)
30–39	81,000	65,816 (81.3)	65,373 (80.7)	42,622 (52.6)	4,394 (5.4)
40–49	102,000	86,766 (85.1)	86,378 (84.7)	63,823 (62.6)	7,628 (7.5)
50–59	92,000	80.194 (87.2)	79,928 (86.9)	67,359 (73.2)	10,996 (12.0)
60–64	39,000	35,579 (91.2)	35,492 (91.0)	32,646 (83.7)	14,551 (37.3)
65+	188,000	175,366 (93.3)	174,813 (93.0)	167,136 (88.9)	122,420 (65.1)

The table shows the number of persons vaccinated by vaccine dose and by age group who were entered into the official Vaccination Record System as of 16 September 2022. The total population covered is based on the resident population as of 1 January 2022 and age categories are based on age as of 1 January 2022.
